# Period poverty: menstrual health hygiene issues among adolescent and young Venezuelan migrant women at the northwestern border of Brazil

**DOI:** 10.1186/s12978-021-01285-7

**Published:** 2021-11-27

**Authors:** Rachel E. Soeiro, Leila Rocha, Fernanda G. Surita, Luis Bahamondes, Maria L. Costa

**Affiliations:** Department of Obstetrics and Gynecology, Faculty of Medical Sciences, The University of Campinas, 101 Alexander Fleming St, Campinas, SP Brazil

**Keywords:** Adolescent/young women, Menstrual health, Period poverty, Migrant, Venezuela, Brazil

## Abstract

**Background:**

Adolescent and young women (10–24 years old) are habitually a neglected group in humanitarian settings. Menstrual hygiene management (MHM) is an unmet aspect of sexual and reproductive health (SRH) and an additional challenge if lack of hygiene products, inadequate access to safe, clean, and private toilets identified as period poverty. Our objective was to provide an overview of the main MHM issues affecting Venezuelan migrant adolescents and young women in the north-western border of Venezuela-Brazil.

**Method:**

A cross-sectional study was conducted, early in 2021, with the use of a self-responded questionnaire, in Spanish, adapted from the Menstrual Practice Needs Scale (MPNS-36). All identified adolescents and young women aged between 12 and 24 years old were invited to participate (convenience sample-167 women). Women with complete questionnaires and who menstruate were included. Information on access to and quality of hygiene kits and toilets were retrieved, and a descriptive analysis performed, with an evaluation of frequencies for categorical variables (n, %) and mean (± SD-standard deviation) for continuous variables. In addition to the open-ended questions, we included one open question about their personal experience with menstruation.

**Results:**

According to official reports, at the moment of the interviews, there were 1.603 Venezuelans living on the streets in Boa Vista. A total of 167 young women were invited, and 142 further included, mean age was 17.7 years, almost half of the participants who menstruate (46.4%) did not receive any hygiene kits, 61% were not able to wash their hands whenever they wanted, and the majority (75.9%) did not feel safe to use the toilets. Further, menstruation was often described with negative words.

**Conclusions:**

Migrant Venezuelan adolescents and young women have their MHM needs overlooked, with evident period poverty, and require urgent attention. It is necessary to assure appropriate menstrual materials, education, and sanitation facilities, working in partnership among governmental and non-governmental organizations to guarantee menstrual dignity to these young women.

## Plain language summary

Adolescent and young women (10–24 years old) are habitually a neglected group in humanitarian settings (situations of forced displacement, armed conflict, or natural disaster) and, in those contexts, they hardly have access to hygienic menstrual products, safe toilets, or water. This study provides an overview of the menstrual hygiene management issues among Venezuelan adolescents and young migrants living in the northwestern Brazilian border. We found almost half of the participants who menstruate (46.4%) did not receive any hygiene kits, 61% were not able to wash their hands whenever they wanted, and the majority (75.9%) did not feel safe to use the toilets evidencing the period poverty (lack of menstrual supplies, private toilets, sanitation conditions, and education) that affects the wellbeing of these women, especially during humanitarian crisis. Knowing about the Venezuelan adolescent migrant’s menstrual health management issues may help other humanitarian settings to discuss and address those needs, reducing the physical, psychological, and social consequences of menstrual poverty.

## Abstract (es)

**Contexto:**

Adolescentes e mulheres jovens (10–24 anos) são frequentemente negligenciadas em contextos humanitários. O manejo da higiene menstrual (MHM) é um aspecto ignorado em saúde sexual e reprodutiva (SSR) e um desafio adicional é a pobreza menstrual: falta de produtos de higiene pessoal, acesso inadequado a banheiros seguros, limpos e privados. Nosso objetivo foi fornecer uma visão geral das principais questões no MHM que afetam adolescentes e mulheres jovens imigrantes venezuelanas na fronteira da Venezuela com o Brasil.

**Método:**

Foi realizado um estudo transversal no qual aplicou-se um questionário autorrespondido, em espanhol, adaptado da “Menstrual Practice Needs Scale” (MPNS-36) em janeiro de 2021, 167 adolescentes e mulheres jovens com idades entre 12 e 24 anos foram convidadas a participar, 142 responderam ao questionário. Os dados obtidos foram inseridos em um Banco de Dados elaborado para o estudo, no programa Excel para Windows e analisados no software SPSS. Foi realizada análise descritiva dos dados, com avaliação de frequências para variáveis categóricas (n, %) e média (± DP-desvio padrão) para variáveis contínuas. Recuperamos as informações de acesso e qualidade dos kits de higiene e realizamos uma análise descritiva. Além das questões de múltipla escolha, incluímos uma questão aberta: “Como é a menstruação para você?”.

**Resultados:**

Segundo informações oficiais, no momento das entrevistas, havia 1.603 venezuelanos vivendo nas ruas de Boa Vista. Foram entrevistadas 142 adolescentes, com média de idade de 17,7 anos, quase metade das participantes que menstruavam (46,4%) não receberam kit de higiene, 61% não conseguiam lavar as mãos quando desejassem e a maioria (75,9%) não se sentia segura para usar o banheiro. Além disso, a menstruação foi frequentemente descrita com palavras negativas.

**Conclusões:**

Adolescentes e mulheres jovens imigrantes venezuelanas têm suas necessidades no MHM negligenciadas, com evidente pobreza menstrual, e requerem atenção urgente. É necessário garantir absorventes, educação e saneamento básico, trabalhando em parceria entre organizações governamentais e não governamentais para garantir a dignidade menstrual a essas jovens.

## Background

It is estimated that there are at least 79.5 million people worldwide who have left their homes due to armed conflict, persecution, generalized violence, lack of economic opportunities, or human rights violations [1]. Around half of them are adolescent girls and women of reproductive age [1, 2]. The World Health Organization (WHO) defines adolescents as the group aged from 10 to 19 years old [3]. Nevertheless, research concerning adolescents is often extended to include individuals until 24 years old, defined as young adults or Youth, in agreement with contemporary patterns of adolescent growth [4].

Adolescent girls and young women are a neglected group in humanitarian settings [5] and their sexual and reproductive health (SRH) issues are habitually neglected [6]. They have limited knowledge about contraceptive methods, sexually transmitted infections (STIs), and are more vulnerable to unplanned pregnancies, leading to increased rates of unsafe abortion, maternal morbidity, and mortality. Gender-based, domestic, and sexual violence is also a key concern among this group [6, 7]. Issues considered trivial in other contexts, are of relevance in vulnerable populations, with important impact on individual wellbeing and healthcare. Menstrual hygiene management (MHM) is an unmet aspect of SRH and can be an additional challenge for displaced adolescents and young women, due to a period poverty: lack of hygiene products, inadequate access to safe, clean, and private toilets that all of them impacting in their health and wellbeing [8–10]

In Latin America, the Venezuelan economic crisis during the last 5 years, led that almost 5.4 million Venezuelans leave the country [11], and it is considered the largest displacement in the history of the region [1, 12]. It was estimated that since 2017 over 455,000 Venezuelans have arrived in Brazil, and of these, about 40,000 currently reside in the city of Boa Vista (the state capital, near to Venezuelan border), representing around 10% of the local population [12, 13]. The Brazilian Government, in collaboration with the United Nation High Commissioner for Refugee (UNHCR), built 13 shelters in the state hosting at early May 2021, 7,175 Venezuelans as transit location waiting for a definitive resettlement in other parts of the country [14].

Due to the SARS-CoV-2 (COVID-19) pandemic, since March 2020, the Brazilian border with Venezuela was closed [15]; nevertheless, the Venezuelans continue to cross the border through alternative routes [16, 17]. The International Organization for Migration (IOM) reported around 1603 Venezuelans living in tents behind the Roraima’s bus station, including 84 female adolescents (12–17 years old) [16]. Further, MHM is an important issue for migrant women worldwide [4]. Due to the scarce information regarding Venezuelan migrant adolescents about MHM, our aim was to provide an overview of the main MHM issues affecting migrant Venezuelan adolescents and young women in Boa Vista, Roraima, Brazil.

## Methods

### Study design and study tools

A cross-sectional study was conducted, with the use of a self-responded questionnaire, designed for this study, adapted, and translated into Spanish (the native language of the Venezuelan) from the Menstrual Practice Needs Scale (MPNS-36) [18]. This scale was developed after a literature review about menstrual practices in low-and middle-income countries and was assessed in a pilot survey in Uganda. It is available for download and can be further adapted for different ages and contexts [18, 19]. The questionnaire used in the current study included one open question about menstruation and multiple-choice questions on sociodemographic characteristics (age, ethnicity, cohabitation status, years of schooling, employment, income, place of residence, and migration information), access to and quality of hygiene kits, and toilets. A unique pre-defined identification to each adolescent and young woman was attributed, respecting data confidentiality.

### Study participants and sampling

Since 2019, there are hundreds of Venezuelans living in tents behind the Boa Vista bus station. The Brazilian army is responsible for organizing the daily routine at the place, providing food, vaccines, and some hygiene kits. In this place, there are limited non-potable water points among the tents, one place used as restroom, with toilets and showers, and one point adapted for laundry. It is estimated that 1603 Venezuelans non-legally documented are living in that place, including 84 adolescent girls (12–17 years old) [16].

A sample was selected from Venezuelan adolescents and young women living in tents behind the Boa Vista bus station, however those living in UNHCR shelters or in informal non-UN settlements in Boa Vista who attended the St. Agostinho church, a location that provides food and other essential items that migrant needed including hygiene kits under a program managed by UNICEF and CARITAS International, were also included.

A total of 167 adolescents and young women were invited to participate, 153 completed the questionnaire and 142 reported menstruation.

### Data collection

The study was conducted in Boa Vista, capital of Roraima state. Due to the epidemiological condition of the Covid-19 pandemic, the research team was not authorized to enter the UNHCR shelters, as initially established and the only allowed places to perform the interviews were informal shelters. The largest one, located at the Boa Vista bus station and at the St. Agostinho church.

Two female healthcare providers (one physician and one nurse) were responsible for the data collection between 18 and 23 January 2021. The team identified young women between 10 and 24 years old (fluent in Spanish and literate) at the informal shelter or the St. Agostinho church and further invited them to participate in the study. A self-responded questionnaire was applied with an average duration of 30 min. Participants did not receive financial compensation.

### Statistical analysis

A descriptive analysis was performed, the simple distribution was initially performed for numeric variables (using frequency, means, and standard deviations (SD), range, median, and quartiles). There was one open question in the questionnaire, on the women’s personal experience/feeling about menstruation, as: “*How is menstruation for you?”* The answers were grouped by the frequency of the most used words and analyzed by similarity considering a keyword, from that, a visual representation of the results was created.

### Ethical issues

The study protocol was approved by the Ethics Committee of the University of Campinas, Brazil and all participants signed an informed consent or assent form prior to being interviewed (IRB no. 20458219.0.0000.5404).

For the unaccompanied girls under 18 years of age, a waiver for the need that a responsible adult signed the informed consent form was obtained. The Brazilian regulation for research involving human beings [20] only accepted research without the consent signed for the legal responsible, in case of vulnerable populations [20], understanding that it could add an additional risk for the adolescents when considering questions on SRH issues. Nevertheless, all included young women have signed an assent form and were exhaustively elucidated about the research. The UNHCR and the Roraima State Underage Guardianship Council (Roraima’s Conselho Tutelar) also authorized the study prior to its implementation.

## Results

A total of 167 adolescents and young women were invited to participate, 142 (12–24 years old; 85.0%) completed the questionnaire; the age (mean ± SD) was 17.7 (± 3.6). Ten adolescents aged 10–11 years old were excluded because they did not adequately respond, filling out all alternatives in every question, invalidating their analysis and other four (under 18 years old), 11 adolescents were excluded because they reported that they had not yet had menarche and other four (under 18 years old), were also excluded because they were deprived of authorization by their mothers to participate in the study due to the topic related to sexual and reproductive health issues, including menstruation. In relation to housing conditions, the majority (84.5%) was living on the tents behind the Roraima’s bus station, and for most of them (80%) the main source of income since they arrived in Brazil, was donations (Table 1).

**Table 1:** Sociodemographic characteristics of the migrant Venezuelan adolescents and young women interviewed at the Brazilian-Venezuelan border (n = 142), 2021^a^

Characteristics of the adolescent (n = 142)	N	(%)
Age (years)		
10–19	83	58.4
20–24	59	41.6
Adolescent younger than 18 years old unaccompanied?^b^ (n = 81)		
Yes	28	34.6
No	53	65.4
Race (n = 142)		
White	34	23.9
Biracial	60	42.3
Black	24	16.9
Asian	24	16.9
Schooling (n = 142)^c^		
0–4 years	46	32.4
5–10 years	77	54.2
11 or more years	19	13.4
Main reasons for migrating (n = 142)		
Lack of economic opportunities	101	71.1
Insecurity	12	8.5
Hunger	7	4.9
Corruption	5	3.5
Don’t know	17	12.0

^a^Multiple choice questions. Were included all the adolescents and young women who menstruate

^b^In Brazil the legal majority is from the age of 18 years old

^c^Completed years at school. In South American countries, 0–4 years at school, means they did not finish primary school, 5–9 years means they completed primary school and 10 or more years means they studied until secondary school or more. At the moment of the interview, none of the adolescents was studying

**Fig. 1:**
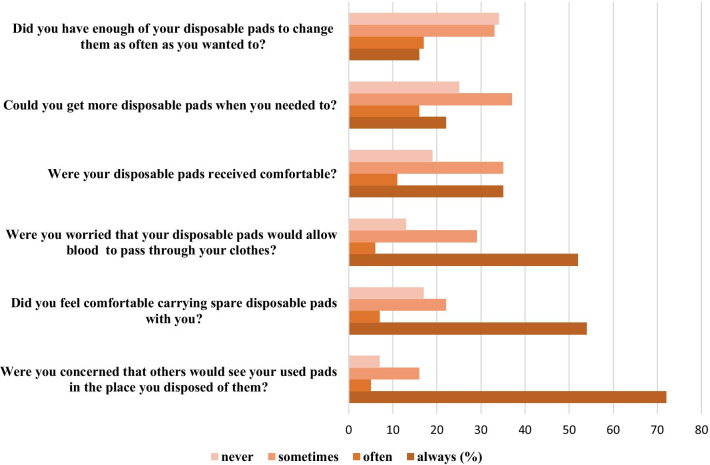
Access to menstrual materials received by the migrant Venezuelan young women at the Brazilian-Venezuelan border. Only the adolescents and young women who received disposable pads answered this section (n = 69)

A half of the interviewed females (50%) who menstruate did not receive any hygiene kit since they arrived at Boa Vista (Table 2). In addition, among those who received disposable pads (45%), little more than a half (53.6%) reported that the pads’ material was rarely or never comfortable and there were not distributed in sufficient quantity (33.3%) (Fig. 1). No menstrual caps were distributed in this population.

**Table 2:** Access to hygiene kits by migrant Venezuelan adolescents and young women interviewed at the Brazilian-Venezuelan border (n = 153), 2021

Since arriving at Boa Vista, which items of hygiene have you received?(n = 153)	N	%
Disposable sanitary pads	69	45.1
Others hygiene items (soap, shampoo, toothpaste, tooth brush)	2	1.3
None	71	46.4
Haven't had menarch yet	11	7.2
**How were hygiene items distributed? (n = 153)**		
International Organization for Migration (IOM)	8	5.2
United Nations Population Fund (UNPF)	5	3.3
Caritas	6	3.9
Brazilian army	8	5.2
Other	6	3.9
Don’t know	37	24.2
Didn’t receive	72	47.1
Haven't had menarch yet	11	7.2
**Where were the kits distribute? (n = 153)**		
Bus station shelter	46	30.1
Caritas Unit	6	3.9
Other	19	12.4
Didn’t receive	71	46.4
Haven't had menarch yet	11	7.2

^a^Were included all the adolescents and young women who menstruate

Concerning the sociocultural concepts about menstruation, one third of the interviewed females do not feel comfortable about carrying pads with them and almost all of them (93.2%) in a certain way were concerned that someone could see the pads in the place where they were disposed (Fig. 1).

**Fig. 2:**
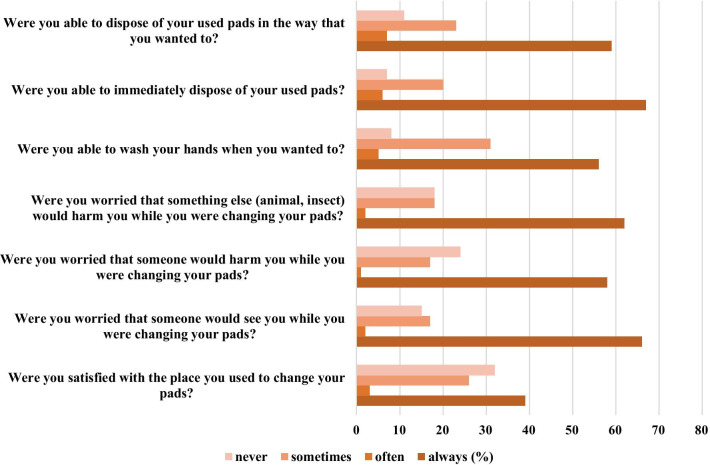
Sanitation conditions regarding MHM among the migrant Venezuelan young women at the Brazilian-Venezuelan border. Were included all the adolescents and young women who menstruate (n = 142)

Although the majority (88%) reported they had access to a toilet for changing pads, these places do not offer adequate sanitation conditions, 61% were not able to wash their hands whenever they wanted and did not feel safe to use the toilets. Most of the interviewed women reported, at least sometimes, they were afraid to be harmed by someone (75.9%) or by an animal or insect (82%) (Fig. 2).

**Fig. 3:**
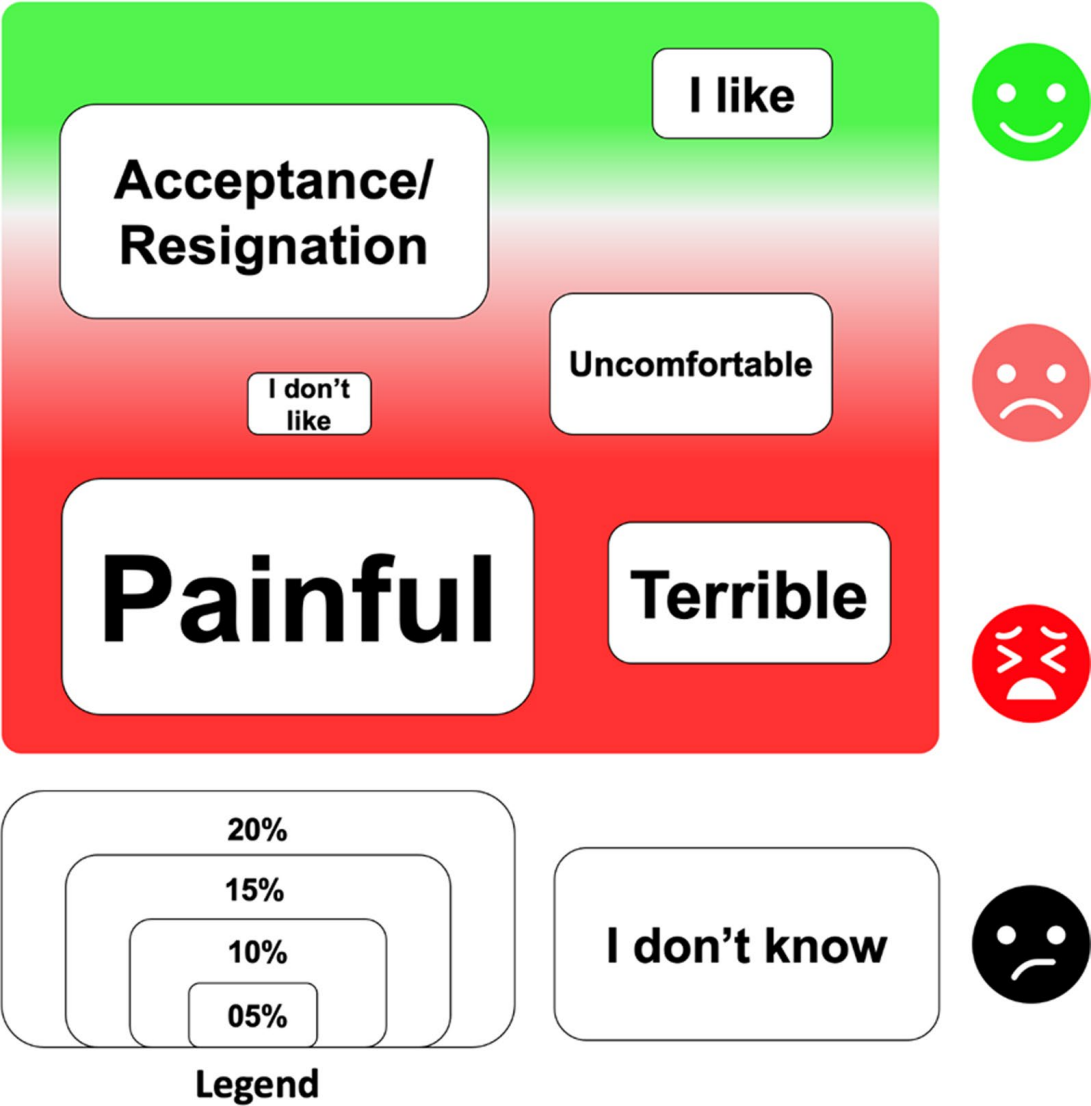
How is menstruation for you? 114 adolescents and young women answered this question

Regarding the open question (“*How is menstruation to you?”*), 28 participants (19.7%) did not respond it. Among those who responded, almost a quarter said they did not know, for the others in general the responses about menstruation were often described with negative words as horrible, terrible, bad, or painful. Figure 3 shows the grouping of words in their meanings according to the frequencies in which they appeared.

## Discussion

Menstrual poverty among the Venezuelan migrant youth in Boa Vista, Brazil was evident: lack of access to adequate menstrual hygiene products, sanitation conditions and toilets.

Less than a half of the interviewed adolescents and young women had received menstrual materials (disposable pads), and for those, one-third reported that the quantity distributed was not enough for a month period. Further, menstrual caps were not allowed in this group of young women. The literature showed that in the lack of appropriate materials, the adolescents and young women handle menstruation with methods that could be unhygienic as reusable old cloth, tissue paper, leaves, wool pieces, or cotton [10, 21, 22]; this could cause discomfort, irritation, and potentially increase the risk of reproductive tract infections (RTI) [22, 23]. In the year 2014, the United Nations Educational, Scientific and Cultural Organization (UNESCO) reported that 1 out of every 10-menstruating youth miss school during their menstrual cycle due to lack of access to menstrual products and resources. Further, in many developing countries many schools do not have sufficient toilets and when exist they were without adequate privacy and in many cases, they provided poor water, sanitation and hygiene infrastructure [24]. If this situation was described in settings not under humanitarian crisis it is possible to imagine what happens among females living almost on the streets as occurred with the interviewed females.

In addition, it was described that those adolescents and young women in humanitarian settings can suffer sexual exploitation trying to manage their MHM needs [10, 22]. In our study, we observed that the majority of females reported fear and anxiety of leakage of bleeding through the clothes which was also reported in other humanitarian contexts causing psychological and social effects as harassment, isolation, and absenteeism at school [9, 10, 25].

Regarding the access to private toilets and sanitation infrastructure, not all the interviewed youth were able to use the toilets and the majority were unable to wash their hands, acknowledging the negligence already reported previously in MHM in emergency settings [8–10]. The absence of adequate sanitation facilities can increase the risk of sexual violence [22, 26] which was related as a fear by the majority of adolescents and young women in our study.

A stigma or taboo about menstruation in this group of migrants is clear, including the finding of mothers who have deprived their daughters to participate in this research, highlighting the characteristics of this transgenerational taboo, which contributes to the persistence of menstrual poverty [27]. Almost 30% of them did not answer the questions or answered that they did not know what menstruation for them is; moreover, for the others, menstruation was associated either with negative feelings or resignation. This has been described in other studies regardless the nationality, religious, or cultural beliefs [9, 10, 22–25], underlying the needs of education on menstrual and reproductive health with the youth and the communities, so that everyone, including men, would be knowledgeable and comfortable in discussing MHM issues [10, 22, 27].

The interviewed females reported a shortage of menstrual supplies, private toilets, sanitation conditions, and comprehensive information. This situation has been published previously in studies with adolescents and young women in other humanitarian contexts including in low- and middle-income countries; with socio-psychological impacts in quality of life and in reproductive health of this population [22–24, 26]. Regarding the reality in Boa Vista, so far, the Brazilian government does not have a policy to address period poverty. On the latest October 07th, Jair Bolsonaro, Brazilian president vetoed a bill that provided for the distribution of sanitary pads to vulnerable populations. [28]

An international effort to alert and educate about this condition was created by the Alliance for Period Supplies: Period Poverty Awareness Week which took place on the last week of May (24–30) 2021 [29]. This initiative is very important to raise society’ awareness, prompting to pressure governments to develop an educational policy that demystifies menstruation and ensures hygiene kits for adolescents, as has been done in Canada, Australia and New Zealand [30].

In humanitarian settings, the presence of other actors as NGO´s, working in collaboration with governments and community leaders is also very important to educate, advocate, provide adequate sanitation installations and assure access to hygiene kits [31].

This study has some limitations. Due to the COVID-19 pandemic it was not possible to have access to the UNCHR shelters, as initially agreed, consequently the results are almost only from migrants living on the streets. Since a self-responded questionnaire was used, adolescents between 10 and 11 years old were excluded because they did not complete the questionnaires adequately. Also, focus group discussions could be more appropriate to enable more data from the younger adolescents. However, the study strength is that, as far as we know, it is the first report which provides an overview of the status of MHM issues among Venezuelan adolescent migrants.

## Conclusions

In conclusion, the migrant Venezuelan adolescents and young women in Boa Vista have their MHM needs overlooked and due to the COVID-19 pandemic they might be more affected since they are living in precarious conditions. Efforts to address the MHM needs from this population require urgent attention.

Despite the COVID-19 pandemic, it is necessary to strengthen the collaboration among NGO’s which are already working in Boa Vista, UNHCR shelters, the Brazilian army and local leaders discussing menstrual health, offering menstrual and hygiene kits, building adequate sanitation with proper water and specific toilets for women and advocating a government policy to address period poverty, in order to guarantee a menstrual dignity to this neglected population.

## Data Availability

The datasets used and/or analyzed during the current study are available from the corresponding author on reasonable request.

## Abbreviations

IOM: International Organization for Migration
IRB: Institutional Review Board
MHM: Menstrual hygiene management
MPNS-36: Menstrual Practice Needs Scale
SARS‑CoV‑2: Severe acute respiratory syndrome coronavirus 2
SRH: Sexual and reproductive health
STI: Sexually transmitted infections
UNESCO: United Nations Educational, Scientific and Cultural Organization
UNHCR: United Nation High Commissioner for Refugee
WHO: World Health Organization
